# Comparative Virological and Pathogenic Characteristics of Avian Influenza H5N8 Viruses Detected in Wild Birds and Domestic Poultry in Egypt during the Winter of 2016/2017

**DOI:** 10.3390/v11110990

**Published:** 2019-10-27

**Authors:** Yassmin Moatasim, Ahmed Kandeil, Basma Emad Aboulhoda, Rabeh El-Shesheny, Maha Alkhazindar, Elsayed Tarek AbdElSalam, Omnia Kutkat, Mina Nabil Kamel, Ahmed Nageh El Taweel, Ahmed Mostafa, Joseph T. Hicks, Sary Khaleel Abd elghaffar, Ghazi Kayali, Mohamed Ahmed Ali

**Affiliations:** 1 Center of Scientific Excellence for Influenza Virus, Environmental Research Division, National Research Centre, Giza 12622, Egypt; yassmin.moatasim@gmail.com (Y.M.); kandeil_a@hotmail.com (A.K.); ra_eny@yahoo.com (R.E.-S.); omnia.abdelaziz@human-link.org (O.K.); minanabil56@yahoo.com (M.N.K.); ahmed.nageh@human-link.org (A.N.E.T.); ahmed_nrc2000@hotmail.com (A.M.); 2 Department of Anatomy and Embryology, Faculty of Medicine, Cairo University, Cairo 11562, Egypt; doctor.basma@hotmail.com; 3 St. Jude Children’s Research Hospital, 262 Danny Thomas Place, Memphis, TN 38105, USA; 4 Department of Botany and Microbiology, Faculty of Science, Cairo University, Gamaa Street, Giza 12613, Egypt; malkhazi@aucegypt.edu (M.A.); tsayed1969@hotmail.com (E.T.A.); 5 Center for Ecology of Infectious Disease, University of Georgia, Athens, GA 30602, USA; joseph.hicks@uga.edu; 6 Pathology and Clinical Pathology Department, Faculty of Veterinary Medicine, Assuit University, Assuit 71526, Egypt; sary64@windowslive.com; 7 Human Link, Hazmieh 1109, Lebanon; 8 Department of Epidemiology, Human Genetics, and Environmental Sciences, University of Texas, Houston, TX 77030, USA

**Keywords:** avian influenza virus, H5N8, Egypt, pathogenicity

## Abstract

The surveillance and virological characterization of H5N8 avian influenza viruses are important in order to assess their zoonotic potential. The genetic analyses of the Egyptian H5N8 viruses isolated through active surveillance in wild birds and domestic poultry in the winter of 2016/2017 showed multiple introductions of reassortant viruses. In this study, we investigated and compared the growth kinetics, infectivity, and pathogenicity of the three reassortant forms of H5N8 viruses detected in wild birds and domestic poultry in Egypt during the first introduction wave in the winter of 2016/2017. Three representative H5N8 viruses (abbreviated as 813, 871, and 13666) were selected. The 871/H5N8 virus showed enhanced growth properties in vitro in Madin Darby canine kidney (MDCK) and A549 cells. Interestingly, all viruses replicated well in mice without prior adaptation. Infected C57BL/6 mice showed 20% mortality for 813/H5N8 and 60% mortality for 871/H5N8 and 13666/H5N8, which could be attributed to the genetic differences among the viruses. Studies on the pathogenicity in experimentally infected ducks revealed a range of pathogenic effects, with mortality rate ranging from 0% for 813/H5N8 and 13666/H5N8 to 28% for 871/H5N8. No significant differences were observed among the three compared viruses in infected chickens. Overall, different H5N8 viruses had variable biological characteristics, indicating a continuous need for surveillance and virus characterization efforts.

## 1. Introduction

Avian influenza viruses (AIVs) infect a wide range of avian species including domestic poultry and wild birds and exist in high pathogenic (HPAI) or low pathogenic (LPAI) forms [1,2]. HPAI viruses cause systemic infection and high mortality in poultry species and belong to either the H5 or H7 hemagglutinin (HA) subtypes. Since the detection of HPAI H5N1 subtype in 1996, the virus has evolved into 10 genetically defined clades (0 to 9) and numerous subclades based on the evolution of the hemagglutinin (HA) gene and has spread through four continents, affecting domestic poultry, wild birds, and mammals. More recently, H5N1 viruses have undergone reassortment with other AIVs and exchanged the N1 segment with other serotypes of neuraminidase to generate different subtypes of H5NX viruses [3,4]. Since late 2014, H5 clade 2.3.4.4 HPAI viruses have had a wide geographic dispersion, with movement from East Asia to North America, West Asia, Europe and Africa. Two distinct groups (group A and group B) of HPAI H5N8 viruses were characterized during massive outbreaks in the period from 2014 to 2016 [5].

Asian-origin HPAI H5N8 viruses emerged in Asian and European countries in 2014 and were isolated from wild birds and poultry [6]. In the winter of 2016, the H5N8 subtype of clade 2.3.4.4 (group B) was reported in migratory wild birds in two Mediterranean regions of Egypt [7,8]. Since then, several H5N8 outbreaks were reported in domestic poultry in several governorates in Egypt [9]. Despite this wide dissemination of H5N8 viruses, there have been no reports of human infection associated with these HPAI H5N8 viruses.

Several introductions of AI H5N8 viruses were detected in healthy, sick, and dead birds in Egypt [10,11]. Sequence analysis of H5N8 viruses showed that all detected viruses were related to clade 2.3.4.4 B. Differences in the genetic makeup of characterized viruses from wild birds and domestic poultry in Egypt showed that H5N8 viruses are genetically heterogeneous, with gene segments coming from different geographical regions [10]. However, the virological and pathobiological characteristics of AI (H5N8) viruses detected in Egypt remain unclear. Here, we compared the growth kinetics, infectivity and pathogenicity of the three reassortant forms of the H5N8 viruses detected in wild birds and poultry in Egypt during their first introduction wave from 2016 to 2017. 

## 2. Materials and Methods

### 2.1. Phylogenetic Analysis of 2.3.4.4 A (H5N8) Egyptian Influenza Virus Genes

The first wave of H5N8 introductions in Egypt was detected through our active surveillance of AIVs in wild birds and poultry between December 2016 and February 2017. Available sequences of HPAI H5 HA genes, NA genes, and internal gene segments from all AIVs from Europe, Central Asia, the Middle East, Sub-Saharan Africa and South Asia collected between 1 January 2005 and 1 May 2017 were downloaded from the Global Initiative on Sharing All Influenza Data (GISAID) (http://platform.gisaid.org/epi3/) and GenBank on 31 July 2017. Each segment was separately aligned with the newly sequenced Egyptian virus genes using Muscle v3.8 [12]. Duplicate sequences were collapsed. Due to the large number of sequences available for each segment (PB2, PB1, PA, NS, NP, MP, HA and NA), a computationally light neighbor-joining tree method (Paup* v4.0) was used to identify genetically distant viral lineages that did not share direct ancestry with the Egyptian H5N8 viruses [13]. These distantly related sequences were removed. Bayesian Evolutionary Analysis Sampling Trees (BEAST) v1.8 was used to investigate the evolutionary relationship of Egyptian H5N8 viruses with closely related AIVs and major ancestral strains available on the GISAID and GenBank. 

### 2.2. Cells

Human lung carcinoma epithelial (A549) cells and Madin Darby canine kidney (MDCK) cells were cultured in Dulbecco’s Modified Eagle’s Medium (DMEM) (BioWhittaker, Lonza, Germany) supplemented with 5% inactivated fetal bovine serum (FBS) (BioWhittaker, Lonza) and 1% antibiotic-antimycotic mixture (BioWhittaker, Lonza) and grown at 37 °C and 5% CO_2_.

### 2.3. Viruses

The plaque-purified HPAI A/northern shoveler/Egypt/813C/2016 (H5N8) (813), A/duck/Egypt/F13666A/2017(H5N8) (13666) and A/green-winged teal/Egypt/871/2016 (H5N8) (871) viruses of the clade 2.3.4.4 were used in this study. The three H5N8 viruses were propagated in allantoic cavities of 11-day-old specific pathogen-free embryonated chicken eggs (SPF-ECEs) for 48 h. The harvests were aliquoted and stored at −80 °C until use. Each virus was titrated using the 50% tissue culture infectious dose assay (TCID_50_/mL) in MDCK cells and the 50% egg infectious dose assay (EID_50_/mL) in SPF-ECEs and titers were calculated by the Reed and Muench method [14].

### 2.4. Growth Kinetics of H5N8 Viruses in Mammalian Cells

Growth kinetics of the three viruses were compared in A549 and MDCK cells. Each virus was inoculated into the two types of cell monolayers at a multiplicity of infection (MOI) of 0.001. The supernatants of the infected cells were collected in triplicates at specific time points (hours post infection (hpi)) and kept at −80 °C. The titer of each collected sample was determined using TCID_50_ and HA assays.

### 2.5. Replication Rate of H5N8 Viruses in SPF-ECEs

The growth properties of the three viruses were compared in SPF-ECEs by inoculating 10^3^ EID_50_ of each virus into 5 SPF eggs and examined for the viability of embryos by candling at each time point. The allantoic fluids were harvested at specific time points and titrated using HA assay and EID_50_/100 µL.

### 2.6. Animal Experiments

Animal experiments were approved by the Medical Research Ethics Committee of the National Research Centre (Ethical permission code: 18040 in April 2018). All animal experiments were conducted in accordance with the recommendations and guidelines of the Egyptian Animal Welfare Legislation. 

### 2.7. Pathogenicity in Mice

Three groups of 11 C57BL/6 mice (6 to 8 weeks old) were anesthetized with isoflurane and intranasally inoculated with 10^6^ EID_50_ in 20 µL of each virus. An uninfected control group was anesthetized and intranasally inoculated with 20 µL of phosphate-buffered saline (PBS). Five mice per group were monitored for 14 days post infection (dpi) for body weight loss and mortality. Mortality was recorded as actual death or loss of  ≥25% of body weight (the threshold at which animals were euthanized). Three mice per group were euthanized at 3 and 6 dpi. Lungs, intestines, and livers were collected. One half of each organ was fixed in 10% formaldehyde and saved at room temperature for histopathology examination. To determine viral titers, 0.1 gm of each organ was homogenized in 0.5 mL PBS with a Qiagen Tissue Lyser II (Qiagen, Hilden, Germany). Organ homogenates were centrifuged at 2000× *g* for 5 min, and the virus titer was determined in the supernatants by TCID_50_/100 µL.

### 2.8. Pathogenicity in Chickens and Ducks

To determine the pathogenicity of the viruses in chickens and ducks, three groups of eleven 4-week-old SPF White Leghorn chickens and 3-week-old Pekin ducks were infected with 100 µL containing 10^6^ EID_50_ of each virus. Infection was done through natural routes (intranasal, intraocular, and intratracheal infection). Five animals per group were monitored for 10 dpi for signs of infection and mortality. Oral swabs, cloacal swabs, and organs (liver, lung, trachea, kidney, spleen, brain, and intestine) were collected from three animals per group at 2 dpi for chickens and at 3, 6, and 9 dpi for ducks. Organs were divided into two parts. The first part was fixed in 10% formaldehyde and saved at room temperature for histopathology examination. The second part was subjected to homogenization as previously described. Swabs and homogenates of organs were subjected to virus titration by EID_50_.

### 2.9. Histopathology

Histopathology was performed on the fixed organs from each viral group to examine the pathological lesions due to viral infection. Histopathological changes in the lung were scored from 0 to 4 as previously described [15]. Briefly, Score 0 was defined as an unremarkable lesion; 1 was scored when there were minimal changes in the bronchiolar epithelium, with minimal peribronchiolar/perivascular inflammation; Score 2 was considered when there were mild multifocal bronchiolar epithelial changes, with perivascular and peribronchiolar inflammation; Score 3 was defined as moderate multifocal bronchiolar epithelial changes, with mild to moderate perivascular, peribronchiolar, and alveolar inflammation; and 4 was defined as marked diffuse bronchiolar epithelial changes, with marked perivascular, peribronchiolar, and alveolar inflammation. Histological criteria used to grade intestinal mucosal lesions and villous damage were classified as previously described [16]: Score 0, normal mucosa; Score 1, focal epithelial cell desquamation; Score 2, more marked epithelial injury with eroded areas; and Score 3, ulceration of the epithelium. The histopathological scoring system for evaluation of hepatic injury was graded on a scale from 0 (no lesions) to 3 (severe lesions) as previously described [17]. Histopathological scoring was performed over 10 randomly selected fields from each animal in the different groups under a magnification of 100. 

### 2.10. Statistical Analysis

GraphPad Prism V5 (GraphPad Inc., La Jolla, CA, USA) was used for statistical analysis. Statistical analysis was performed using one-way ANOVA test, followed by Bonferroni post-hoc testing. Data were represented as mean ± SD. *p* values of ≤0.05 were considered statistically significant. 

## 3. Results

### 3.1. Source of Introductions of Egyptian H5N8 Genes

**Figure 1:**
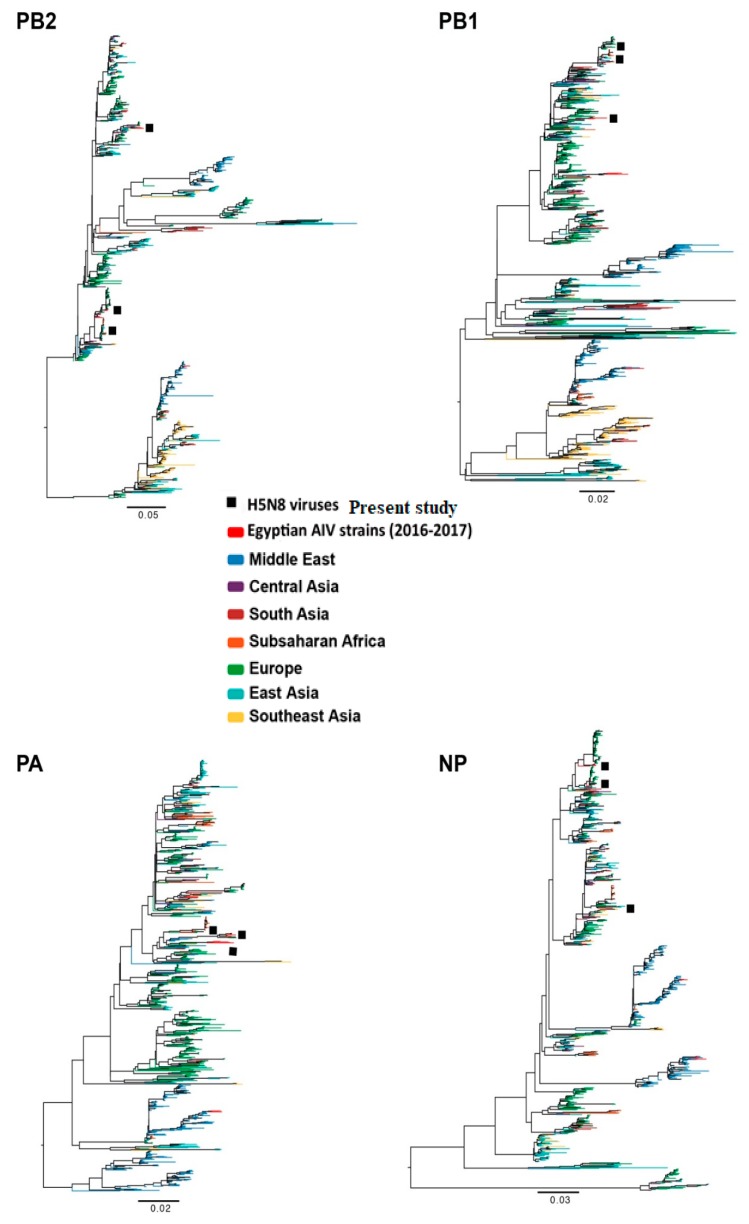

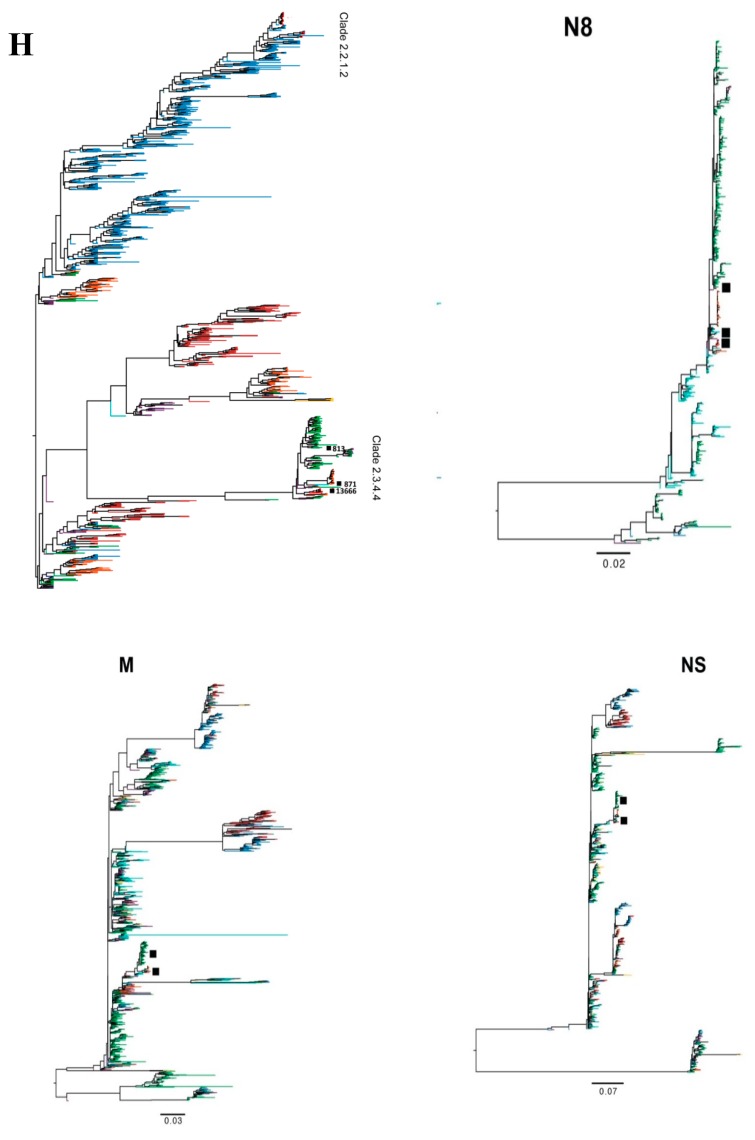
Phylogenetic trees of the nucleotide sequences of the *PB2, PB1, PA, NP, HA, NA, M*, and *NS* genes of the characterized H5N8 viruses in Egypt from domestic poultry and wild birds.

**Figure 2:**
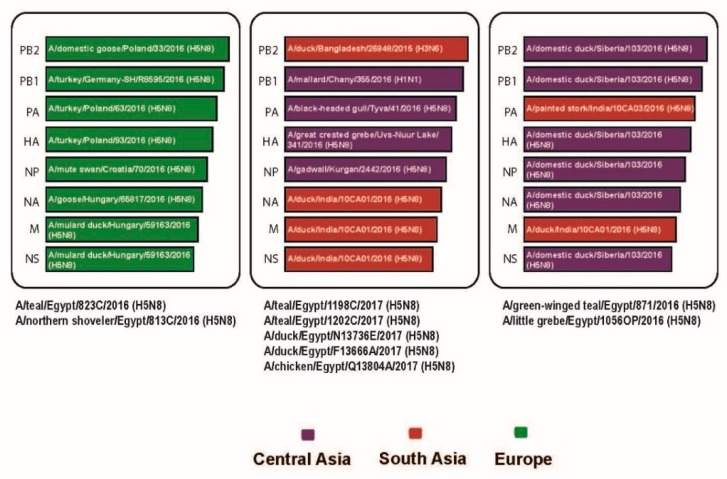
The gnome constellation of the three H5N8 forms detected in poultry and wild birds during our active surveillance study in Egypt. Three forms of H5N8 viruses are represented by triangles containing horizontal bars that represent eight gene segments and the most identical strains.

Analyses of the phylogenetic topologies of eight segments of the detected H5N8 viruses during active surveillance in wild birds and poultry revealed that they were dispersed throughout the phylogenetic trees, indicating at least three independent introductions (Figure 1). Nucleotide Basic Local Alignment Search Tool (BLASTN analysis indicated that three distinct genotypes were present in Egypt and that they were closely related to H5N8 viruses isolated from wild birds and poultry in Europe (Poland, Germany, Hungary, and Croatia), Russia, and Asia (India and Bangladesh) (Figure 2). The similarities of the eight segments among the three forms of Egyptian H5N8 viruses are listed in the Table S1.

The nucleotide sequence similarities of the PB2, PB1, PA, HA, NP, NA, M, and NS segments between the three Egyptian H5N8 strains ranged from 91.8 to 97, 94.5 to 99.3, 95.4 to 98.6, 98.6 to 99, 94.9 to 97.3, 99 to 99.2, 98.7 to 99.1, and 98.7 to 99.1%, respectively. The PB2, PB1, PA, NP, and NA genes diverged into three groups while *M* and *NS* diverged into two groups (Figure 1). 

To identify amino acids that may be involved in virus virulence and mammalian transmission, the deduced amino acid sequences were analyzed for the three forms of Egyptian H5N8 viruses. All Egyptian H5N8 viruses possessed the PLREKRRKR/G at the HA cleavage site, conferring high pathogenicity. Mutations V504 in PB2, V127, L672, and L550 in PA, S64 and P69 in M2, and S42 in NS1 conferring mammalian virulence were detected (Table S2).

Genetic determinants of host range in the PB2, PB1, PA, NP, M1, M2, NS1, and NS2 viral proteins revealed the presence of mammalian preference mutations L13P, K398Q, and G70 in PB1, NP, and NS2, respectively (Table S3). The R57Q mammalian preference determinant in the PA viral protein was detected in the 813 virus. In addition, V667I substitution associated with mammalian preference was detected in the PB2 of the 871 virus. Two variants of PB1-F2 protein were detected that differed in length: 52 residues (2 isolates from wild bird; 871 and 813) and 90 residues (13666 from domestic poultry). Several uncharacterized mutations were detected as shown in Table S4.

### 3.2. Growth Kinetics of H5N8 Viruses in Mammalian Cells and SPF-ECEs

**Figure 3:**
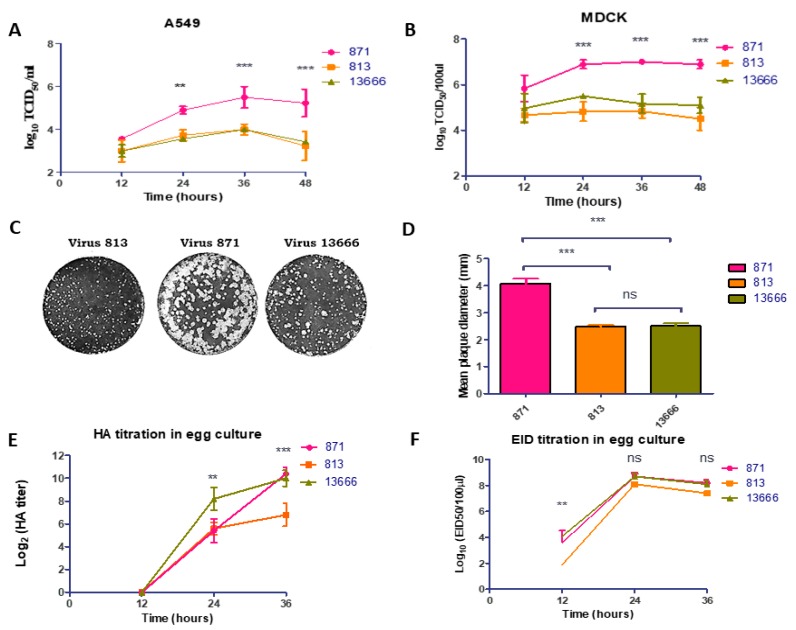
Growth kinetics of the three forms of H5N8 viruses in A549 (**A**), and Madin Darby canine kidney (MDCK) (**B**) cells. The cells were infected with the virus at a MOI of 0.001. At the time points indicated, the supernatant was taken and titrated by 50% tissue culture infectious dose assay (TCID_50_/_mL_) assay on MDCK cells. (**C**) Plaque phenotypes of the three forms of H5N8 viruses on MDCK cells were shown. (**D**) Average plaque sizes were determined from 20 plaques for each virus. Meanwhile, the growth kinetics of the three forms of H5N8 viruses were assessed in embryonated eggs using 10^3^ EID_50_ at 12 hpi. The viral titers in collected allantoic fluids were titrated for their HA assay (**E**) and EID_50_/_mL_ (**F**).

To investigate the growth kinetics of the three different forms of the Egyptian H5N8 AIVs, A549 and MDCK cells were inoculated at an equal MOI of 0.001. The supernatants of the infected cells were collected in triplicates at specific time points and titrated by TCID_50_. No significant difference (*p* > 0.05) was observed between 813 and 13666 in the growth kinetics at different time points. The 871 virus had the highest significant titers at 24, 36 and 48 hpi, when compared to the other viruses (*p* < 0.01) (Figure 3). The plaque morphology of the three viruses was compared in MDCK cells at 72 hpi. The 871 virus had the highest significant plaques size (mean: 4.06 mm in diameter) (*p* < 0.001). While the 813 virus had the smallest plaques with uniformed size (mean: 2.4 mm in diameter), the 13666 virus exhibited a mixed-plaque phenotype, producing both large and small plaques. The plaque sizes reflect the difference in replication rates between the three H5N8 viruses.

To compare the growth properties in SPF-ECEs, the viral titers were assessed at 12 h intervals post inoculation using five SPF eggs per time point. All viruses replicated efficiently in SPF-ECEs. No significant differences were observed among viral HA titers at 12 hpi (*p* > 0.05). Differences in growth kinetics between the three compared viruses were detected at 24 and 36 hpi, when the 13666 virus showed the highest HA titer (*p* < 0.01) at 24 hpi and both 13666 and 871 viruses showed higher HA titer than 813 virus at 36 hpi (*p* < 0.001) (Figure 3). No significant differences were observed among viral EID_50_ titers at 24 and 36 hpi (*p* > 0.05) (Figure 3).

The pathogenicity of the three H5N8 viruses was compared in 15 chicken embryos at different time points. The onset of embryo mortality for the three H5N8 viruses started within 24 hpi. The 13666 strain led to 100% mortality of SPF-ECEs at 30 hpi, while the 871 and 813 H5N8 viruses showed a 60% and 80% mortality rate at the same time, respectively. All embryos were dead at 40 hpi for 871 and 813 H5N8 viruses (Figure S1).

### 3.3. Pathogenicity in Mice

**Figure 4:**
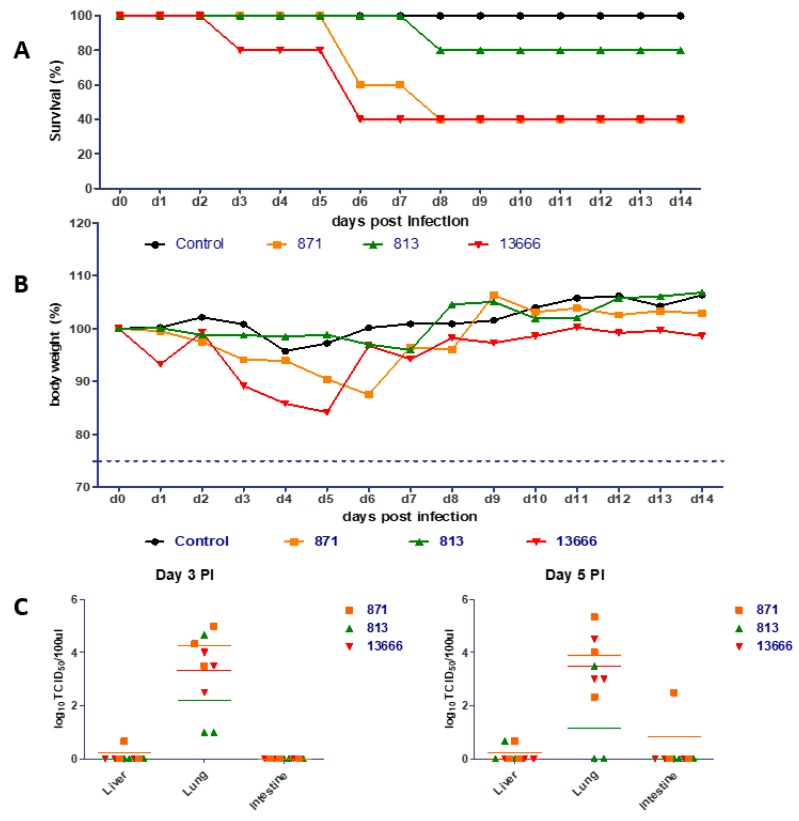
Replication of the three forms of H5N8 viruses in infected mice. C57BL/6 mice were infected with 10^6^ EID_50_ of 871, 813, and 13666 H5N8 viruses. Survival (**A**), change in body weight (**B**), and titers of virus in lungs, intestines and livers at 3 and 5 dpi (**C**) were determined.

The three viruses exhibited differing degrees of virulence in mice at different times of infection (Figure 4A). Mice infected with the 13666 virus showed 20% mortality rate at 3 dpi. The mortality rate increased to the highest rate (60%) at 6 dpi. No deaths were observed among infected mice with 871 and 813 viruses till 6 and 8 dpi, respectively. The 871 virus caused 60% mortality by 8 dpi while the 813 virus caused only 20% mortality by 8 dpi (Figure 4A). 

Notably, mice infected with the 13666 virus experienced rapid weight loss of more than 15% of the body weight during the first five days post infection—lower than observed for the 871 and 813 viruses (Figure 4B). Mice infected with 871 virus demonstrated a loss of more than 12% of the total body weight by 6 dpi. No significant difference of the body weight was observed between the infected mice with 813 viruses and non-infected control group. These results clearly demonstrate the difference in the pathogenicity characteristics of the different Egyptian forms of HPAI A/H5N8 viruses in infected mice. 

### 3.4. Titer in Organs of Mice

Three mice in each group were euthanized on 3 and 5 dpi to determine viral titers in lung, intestine, and liver. The three H5N8 viruses exhibited differing degrees of replication in organs of infected mice. All viruses were detected in the lungs at 3 and 5 dpi (Figure 4C). The 871 AIV titers were markedly higher in lung homogenates of infected mice than were the other viruses at 3 and 5 dpi (Figure 4C). These data indicate that all the three forms of H5N8 replicated in mice without prior adaptation. The intestine of only one mouse of the group infected with the 871 AIV showed 2.5 log_10_ TCID_50_ at 5 dpi, while the 871 and 813 viruses were detected in liver of infected mice at day 5 (Figure 4C).

### 3.5. Viral Replication and Pathogenicity in Ducks and Chickens

**Figure 5:**
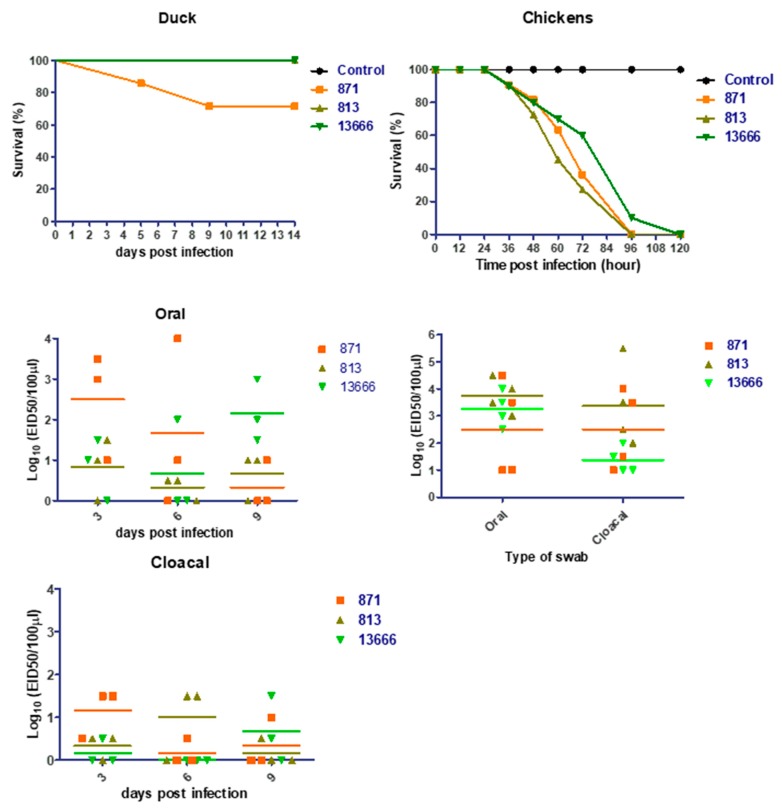
Viral replication and pathogenicity of the three forms of Egyptian H5N8 viruses in ducks and chickens.

None of the ducks infected with 813 and 13666 viruses died till 14 dpi (Figure 5). However, 28% (2 of 7 ducks) of 871 inoculated ducks died (one at 5 dpi, and one at 9 dpi) with obvious disease symptoms such as hemorrhagic skin of feet, discoloration of beak, central nervous system involvement evidence such as incoordination, loss of balance, and motor issues (neurodegenerative disease signs), and secretions from the eyes. Another five ducks infected with the 871 virus showed mild to no signs. Two of seven ducks in each of 813 and 13666 viruses infected groups showed less severe signs with no mortality. The replication rate of 13666 virus in infected ducks was the lowest and virus was detected in different organs in only one of the three infected ducks (Table 1). The virus shedding detected in the collected oral and cloacal swabs of the three infected groups of ducks showed that the 871 virus had the highest viral titer at 3 dpi (Figure 5). Virus shedding continued until 9 dpi (Figure 5). Chickens inoculated with 10^6^ EID_50_ of each of the three H5N8 viruses died from 48 to 96 hpi, with no significant differences among the groups (Figure 5). Typical signs of highly pathogenic infection were observed including ocular and nasal discharges, swelling of the head, listlessness, cyanosis of the unfeathered skin, wattles, and comb, and diarrhea.

The 813 virus was detected from multiple organs and collected swabs of all inoculated chickens with viral titers ranging from 3 to 3.875 EID_50_. The 871 virus was not isolated from spleen and brain of only one of four infected chickens while the remaining organs and collected swabs had viral titer ranging from 2.375 to 3.87 EID_50_. The 13666 virus was detected in all swabs and organs with viral titers ranging from 1.3 to 3.5 EID_50_ except the spleen of two chickens (Table 2).

### 3.6. Histopathology

#### 3.6.1. Lung

Lungs of the uninfected mice were normal. Histopathologic analysis of the lungs of the infected mice revealed that those infected with 871 showed severe interstitial pneumonia invading the lung tissue and destroying the lung alveoli. Furthermore, marked vascular wall degeneration, thrombus formation, irregularity of the bronchioles with degeneration of their lining, intrabronchial cellular debris and inflammatory cells were observed. The 813 virus-infected mice showed diffuse infiltration of the lung with inflammatory cells and erythrocytes. The bronchioles appeared irregularly dilated with disruption of their walls and peri-bronchial inflammatory infiltration. Furthermore, exfoliation of desquamated and inflammatory cells was observed in the lumen of the bronchioles. Some alveoli appeared severely collapsed, others showed compensatory dilatation. The 13666 virus-infected mice showed massive interstitial inflammatory cellular infiltration seen around bronchioles and blood vessels. Severely collapsed alveoli, marked thickening, irregularity, lamellar separation, and degeneration of the blood vessel wall and bronchial epithelial cell desquamation were also observed. 

Histopathologic analysis of the lungs of the uninfected control ducks showed normal lung architecture with polygonal alveoli and intervening blood vessels. The 871 virus-infected ducks showed massive interstitial hemorrhagic pneumonia with nodular inflammatory lymphoid infiltration. The lung parenchyma was completely flooded with inflammatory cells, erythrocytes, and degenerated cells. Rupture of arteriolar walls was observed. Ducks infected with virus 813 showed severe vascular congestion, hemorrhage, and blood extravasation from ruptured vascular wall. Dense lymphocytic infiltration and peri-arteriolar metaplasia were also observed. The 13666 virus caused marked thickening of interalveolar septa and hemorrhage bridging across the lung. The alveolar ducts appear irregularly dilated with erythrocytes inside.

**Figure 6:**
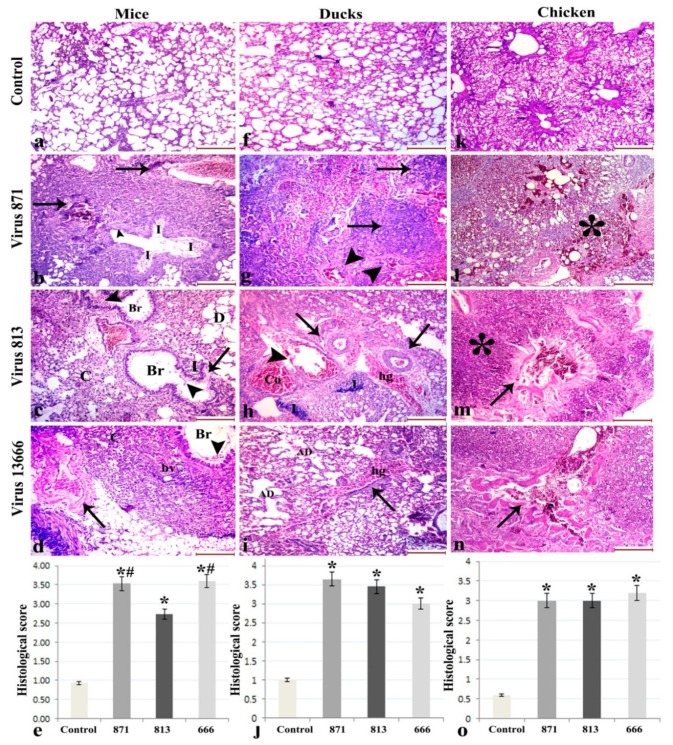
Histopathological changes in lungs of infected mice (**a–d**), ducks (**f–i**) and chickens (**k–n**), with different forms of H5N8 viruses compared with the control animals. Histological scoring of pulmonary lesions of three H5N8 viruses (e,j,o). (*: statistically significant relative to the control group; #: statistically significant relative to virus 813; scale bar 200 µm).(**b**) Infected mice with 871 virus show degeneration of bronchioles lining (arrowheads), inflammatory cells (**I**) and vascular wall degeneration (black arrows). (**c**) 813 infected mice bronchioles (Br) show disruption of their walls (arrow), and inflammatory cells in the lumen (arrowheads). Some alveoli are collapsed (C), while others appear dilated (D). (**d**) Virus 13666 shows massive inflammatory infiltration around bronchioles (Br) and blood vessels (bv), degeneration of the blood vessel wall (arrow) and bronchial epithelial cell desquamation (arrowhead) and severely collapsed alveoli (**c**). (**g**) 871-infected ducks show inflammatory lymphoid infiltration (arrows) and rupture of arteriolar walls (arrowheads). (h) 813 shows severe vascular congestion (Co), hemorrhages (hg) and blood extravasation from ruptured vascular wall (arrowhead), dense lymphocytic infiltration (**I**) and peri-arteriolar metaplasia (arrows). (**i**) Virus 13666 shows thickening of interalveolar septa (arrows), hemorrhage (hg) and irregularly-dilated alveolar ducts (AD). In chickens, 871, 813 and 13666 viruses show inflammatory lymphoid infiltration (asterisk) with degeneration in the vessel wall (arrow).

The chicken control group showed normal lung architecture with normal alveoli and tertiary bronchioles. Lungs of chickens infected with 871 virus showed massive interstitial hemorrhagic pneumonia with inflammatory lymphoid infiltration, while those infected with 813 virus showed severe interstitial pneumonia with diffuse inflammatory lymphoid infiltration associated with degeneration in the vessel wall. The 13666 virus caused marked interstitial pneumonia, with diffuse inflammatory lymphoid infiltration and massive destruction of the vascular wall (Figure 6).

#### 3.6.2. Liver

Histopathologic analysis of the livers of the uninfected control mice showed normal hepatocytes radiating from the central vein. Virus 871-infected mice showed focal areas of subcapsular hepatocellular necrosis together with congestion of the blood vessels. Virus 813-infected mice showed massive areas of hemorrhagic infarctions in the liver parenchyma, with edema in the space of Disse. Virus 13666 caused vascular congestion with perivascular mononuclear cellular infiltration.

Uninfected ducks showed normal hepatic lobular architecture (Figure 6g), Virus 871 caused areas of hepatocellular degeneration, hemorrhagic necrosis, and marked dilatation of the central vein. Virus 813-infected mice showed disruption of the hepatic architecture with vacuolar degeneration of hepatocytes. Mice infected with virus 13666 showed perivascular mononuclear cellular infiltration. 

**Figure 7:**
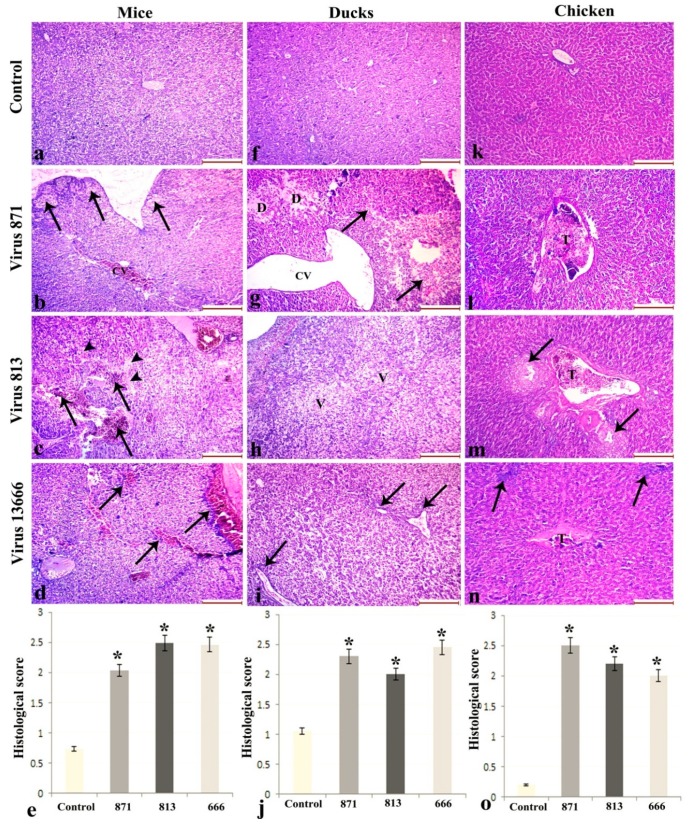
Histopathological changes in liver of infected mice (**a–d**), ducks (**f–i**) and chickens (**k–n**) with different forms of H5N8 viruses compared with the control animals. Histological scoring of intestinal lesions (e,j,o). (*: statistically significant relative to the control group). (**b**) 871-infected mice show hepatocellular necrosis (arrows) and congestion of the central vein (CV). (**c**) 813 shows hemorrhagic infarctions (arrows) with edema in the space of Disse (arrowheads). (**d**) 13666 shows mononuclear cellular infiltration (arrows). In ducks, Virus 871 shows (**g**) hepatocellular degeneration (D), hemorrhagic necrosis (arrows) and marked dilatation of the central vein (CV). (h) 813 shows vacuolar degeneration of hapatocytes (V), and (**i**) 13666 shows mononuclear cellular infiltration (arrows). In chickens, (**l**) Virus 871 shows thrombus formation (T), while (**m**) 813 shows bile duct hyperplasia (arrows) with thrombus formation (T), and (**n**) 13666 shows thrombus formation (T) and focal lymphoid cell reaction (arrows). (Scale bar 200 µm).

Uninfected chickens showed normal hepatic lobular architecture. Chickens infected with 871 showed edema in the Disse spaces and extensive necrosis of the vascular wall with thrombus formation. Those infected with virus 813 showed bile duct hyperplasia, degeneration of the vascular wall with thrombus formation, and mononuclear lymphoid cell reaction while those infected with virus 13666 showed mild degeneration in hepatocytes, degeneration in the vascular wall with thrombus formation, and focal lymphoid cell reaction (Figure 7).

#### 3.6.3. Intestine

Uninfected mice showed normal finger-like intestinal villi. Mice infected with virus 871 showed marked atrophy and attenuation of the villi, with disintegration of lamina propria. Lymphocytic infiltration in the connective tissue core of intestinal villi was also observed.

Uninfected ducks showed a normal intestinal villous structure, with a core of connective tissue, healthy epithelium, intact brush border, and few goblet cells. Ducks infected with virus 871 showed disorganization of the connective tissue core, shedding of the villous epithelium, and loss of intestinal brush border. Virus 813-infected ducks showed shortened denuded intestinal villi with marked increase in goblet cells in the intestinal villi and in the crypts. Virus 13666-infected ducks showed thickening and broadening of the villi, marked disruption of the villus architecture, and aggregation of the goblet cells at the tip of the villus.

**Figure 8:**
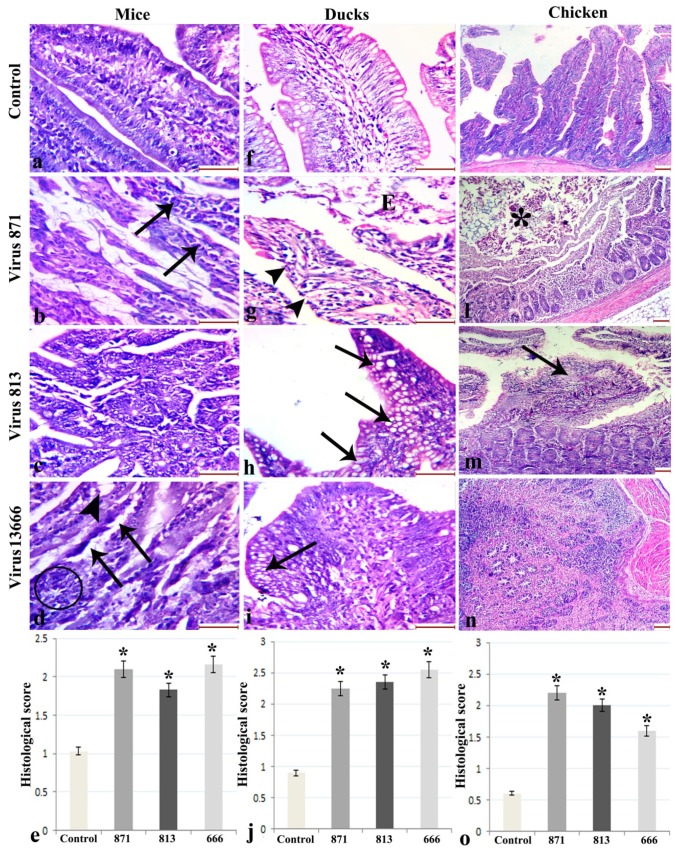
Histopathological changes in intestine of infected mice (**a–d**), ducks (**f–i**) and chickens (**k–n**) with different forms of H5N8 viruses compared with the control animals. (e,j,o) Histological scoring of intestinal lesions (*: statistically significant relative to the control group, scale bar 200 µm). In mice, (**b**) Virus 871 shows Lymphocytic infiltration (arrows). (**d**) Virus 13666 shows subepithelial Gruenhagen’s spaces at the tip of the villi (arrowhead), epithelial lifting down the side of the villus (arrows) and lymphocytic infiltration (circle). In ducks, (**g**) Virus 871 shows shedding of the villous epithelium (E) and loss of intestinal brush borer (arrowheads). (**h**) Virus 813 and (**i**) 13666 shows marked increase in goblet cells (arrows). In chickens, Virus 871 shows necrotic tissue in the intestinal lumen (astrisk), while 813 shows sloughing of the intestinal epithelial lining (arrow).

Uninfected chickens showed normal intestinal villous structure with a core of connective tissue, healthy epithelial covering, and intact brush border. Chickens infected with virus 871 showed severe necrosis of the mucosal layer including the epithelium, lamina propria, and muscularis mucosa. Necrotic tissue was seen in the intestinal lumen. Those infected with virus 813 showed thickening and broadening of the villi, with sloughing of the intestinal epithelial lining. Virus 13666 caused diffuse lymphocytic cellular reaction (Figure 8).

#### 3.6.4. Other Organs

Three HPAI H5N8 viruses caused systematic infection in both poultry species and spread to spleen, trachea, kidney and brain of ducks (Supplementary Figure S2) and chickens (Supplementary Figure S3), causing histopathological changes.

## 4. Discussion

The surveillance and characterization of emerging influenza viruses in wild birds and domestic poultry are the best approaches to monitor the zoonotic potential of emerging viruses. Several outbreaks of HPAI H5N8 virus of the clade 2.3.4.4 were reported in wild birds and domestic poultry in Egypt during the winter of 2016/2017 [7,18]. This clade of HPAI viruses is actively evolving by reassortment with other avian influenza viruses.

The genetic analyses of the Egyptian H5N8 isolates showed at least three introductions of the virus based on the nucleotide sequences of the isolates from both wild birds and domestic poultry. The 871 reassortant H5N8 virus showed enhanced growth properties in vitro in both MDCK and A549 cells. No difference in the growth properties was observed between 813 and 13666 reassorted H5N8 viruses in mammalian cells. The presence of substitution S71Y in PB1-F2 of the 813 virus was previously reported as a mutation causative of very low protein stability [19]. The presence of N66, Y71 and the truncation in the PB1-F2 could explain the low propagation rate of 813 virus in MDCK and A549 cells and justify the relatively small plaques. Further studies will be required to identify the molecular markers that distinguish the growth properties of 871 H5N8 virus in mammalian cells. 

The virulence of AIVs has been linked to the presence of multibasic amino acids at the cleavage site of the HA [20]. The HA of the Egyptian forms of H5N8 viruses possess fewer multibasic amino acids at the cleavage site PLREKRRKR/GLF than the contemporary circulating H5N1 of clade 2.2.1.2 which has one additional basic amino acid upstream of the furin recognition site (PQGEKRRKKR/GLF). Reducing the number of basic amino acids at the cleavage site motif significantly reduced the pathogenicity of H5N1 in mice [21]. In addition to the HA gene, internal genes also contribute to the pathogenicity of AIVs in mammalian and avian hosts [22].

The comparison of the pathogenesis of H5N8 viruses in mice and ducks strikingly revealed a difference. Studies on the pathogenicity in experimentally infected ducks showed a range of pathogenicity markers with mortality rate ranging from 0% (813 and 13666) to 28% (871). Previous studies showed that domestic and wild ducks infected with H5N8 clade 2.3.4.4 showed mortality rates between 0% and 20% [23,24,25]. Our data clearly demonstrated that ducks infected with the three forms of Egyptian H5N8 suffered from a systemic infection with remarkable difference in viral shedding in oral and cloacal swabs and viral spreading in several organs. Our results are consistent with previous pathogenicity studies of clade 2.3.4.4 H5N8 viruses [26,27]. We observed little systemic spread of the 13666 virus exclusively through either pulmonary or extrapulmonary organs. No significant differences were noted among the compared Egyptian H5N8 viruses in infected chickens with the same viral titer. Although the detected HPAI H5N8 are avian viruses, molecular analysis among the three reassorted H5N8 viruses showed virulence or mammalian preference genetic markers as shown in Tables S2 and S3. The substitution V667I detected in the *PB2* gene of the 871 virus was previously known as a marker for increasing virulence and enhancing transmission to humans [28].

Interestingly, H5N8 viruses in Egypt have some mutations that are suspected to be acquired in other influenza virus subtype during virus adaptation from birds to mammals. The 871 virus has 105M in the NP, previously detected during serial passaging of the AI H9N2 virus in mice lung [29]. Presence of distinguishable 356I in the PB2 and 208A in the PA of the 871 virus, 208K in M1 of the 813 virus, and 24L in NA, and 125D in NS1 of the three H5N8 viruses [29,30,31], could explain why H5N8 avian viruses grow to relatively high titers in mice lung without prior adaptation. Thus, it is essential to investigate host-specific markers in diverse virus strains in different hosts and study their virulence to determine the potential of H5N8 viruses for adaptation and virulence in other mammals including humans.

Two lengths of PB1-F2 protein were detected in the Egyptian H5N8 viruses. A full PB1-F2 with 90 residues was detected in the 13666 virus, while PB1-F2 with 52 residues was characterized in both 871 and 813 viruses as a result of a deletion of the 39 amino acids at the N terminal. The PB1-F2 has a definite role in inducing inflammatory response and enhancing polymerase activity and prolonged virus shedding in infected animal models. The presence of serine at position 66 of PB1-F2 was characterized in the 13666 virus and proven by previous studies to have a role in increasing viral virulence in mice but not in ducks [32,33,34] and to be correlated with the early inhibition of IFNs [35,36,37]. Both 813 and 871 viruses have low virulent genetic marker (asparagine) at position 66 of PB1-F2. Full-length PB1-F2 and the presence of S66 could explain why the 13666 virus caused the highest loss in body weight in infected mice. Full PB1-F2 in the 13666 virus also explains the prolonged shedding in duck swabs when compared to 871 and 813. 

Interestingly, the Egyptian H5N8 replicated well in lungs of infected mice without prior adaptation, which could be explained by the presence of amino acid residues L62, R79 in PB1-F2 protein contributors to cytokine release and inflammatory responses [38], P13 in PB1 [39], and G70 in NS2 [28,40].

C57BL/6 mice infected with H5N8 viruses showed between 20% mortality (for 813) and 60% mortality (871 and 13666), which could be attributed to the presence of the NS1 PDZ binding motif 227GESV230 correlated with pathogenicity [28,41] and the PB1-F2 M46T mutation correlated to increased mortality in mice in 871 and 13666 viruses [28], while the 813 NS1 protein is terminated at position 219 due to mutation of the stop codon and possessing M at the PB1-F2 64 site.

The diminished severity of Egyptian H5N8 avian influenza viruses in mammals could be explained by the presence of only one (L75) of the two sites (L75 and Q69) required for PB2-F1 mitochondrial localization [42], the presence of S82 instead of L [38], and the presence of 68TQG70 instead of 68IVF70 cytotoxic sequence in PB1-F2 [19,38] in addition to the presence of the PB2 627E avian preference factors [43] and NP F464-L466 that cause a specific packaging defect affecting the incorporation of segments 3 and 5 [44]. Histopathological changes caused by different H5N8 strains support the previous reports elucidating the ability of the H5N8 virus to replicate well in different organs causing necrotic lesions [45,46]. However, inflammatory reactions varied significantly in different experiments and birds.

Emerging influenza viruses pose a continuous threat to animal and public health. Understanding the ability of a novel AIV to infect and cause disease in mammals and determining its virological characteristics are of utmost importance. The results presented here provide valuable data characterizing the three introductions of H5N8 viruses of clade 2.3.4.4 isolated in Egypt. Our results showed that these viruses are moderately pathogenic in ducks and mice with obvious differences.

Although the avian H5N8 virus was not detected so far in humans, we should remain aware of the potential of this virus to be transmitted from the avian host to the human population. We suggest using the ferret infection model together with the human lung explant culture model for the routine evaluation of all new HPAIV strains.

## Figures and Tables

**Table 1:** Viral titer of the Egyptian H5N8 avian influenza viruses in different organs of infected ducks ^a^.

	Virus	871	813	13666
Organ				
Liver		2 (1/3) ^b^	1 (1/3)	1.5 (1/3)
Spleen		2.25 ± 0.35 (2/3)	1.5 ± 0 (2/3)	1.5 (1/3)
Intestine		2 ± 0 (3/3)	1.33 ± 0.3 (3/3)	1 (1/3)
Kidney		1.5 ± 0 (3/3)	1.5 ± 0 (3/3)	1 (1/3)
Lung		2 ± 0.7 (2/3)	1.5 ± 0 (2/3)	1.5 (1/3)
Trachea		1.16 ± 0.6 (3/3)	1.7 ± 0.8 (3/3)	1 ± 0.7 (2/3)
Brain		1.75 ± 0.35 (2/3)	1.7 ± 0.3 (3/3)	1.5(1/3)

^a^ Data are the mean Log_10_ EID_50_/100 µL ± SD for positive samples. ^b^ Values in parentheses are the number of positive ducks out of each infected group.

**Table 2:** Viral titer of the three forms of Egyptian H5N8 avian influenza viruses in different organs of infected chickens ^a^.

	Virus	871	813	13666
Organ				
Liver		2.5 ± 0.8 (3/4) ^b^	3.375 ± 0.25 (4/4)	2.875 ± 0.25 (4/4)
Spleen		3.875 ± 1.7 (3/4)	3 ± 0.57 (4/4)	2 ± 1.4 (2/4)
Intestine		3.5 ± 0 (3/4)	3.5 ± 0 (4/4)	3 ± 0 (4/4)
Kidney		3.625 ± 0.25 (4/4)	3.5 ± 0 (4/4)	3.5 ± 0 (4/4)
Lung		3 ± 1.3 (4/4)	3.875 ± 0.25 (4/4)	2.75 ± 0.5 (4/4)
Trachea		3 ± 1.3 (4/4)	3.5 ± 0 (4/4)	2.625 ± 0.75 (4/4)
Brain		3.1 ± 1.04 (3/4)	3.5 ± 0 (4/4)	3.5 ± 0 (4/4)

^a^ Data are the mean Log_10_ EID_50_/100 µL ± SD for positive samples. ^b^ Values in parentheses are number of positive ducks out of each infected group.

## Supplementary Materials

The following are available online at www.mdpi.com/xxx/s1; Figure S1: Mortality rate of embryos of SPF-ECE infected with different H5N8 viruses; Figure S2: H&E-stained sections of trachea, kidney, spleen, and brain of infected ducks with different H5N8 viruses; Figure S3: H&E-stained sections of trachea, kidney, spleen, and brain of infected chickens with different H5N8 viruses; Table S1: Nucleotide sequence homology of the 3 forms of Egyptian H5N8 viruses; Table S2: Analysis of mammalian virulence determinants in the viral PB2, PB1, PA, NP, M2, NS1, and NS2 proteins; Table S3: Analysis of host range genetic determinants in the PB2, PB1, PA, NP, M1, M2, NS1, and, NS2 proteins in H5N8 viruses.; Table S4: Amino acid variations among the 3 forms of Egyptian H5N8 viruses.
