# Supplementary material for: Comparative Virological and Pathogenic Characteristics of Avian Influenza H5N8 Viruses Detected in Wild Birds and Domestic Poultry in Egypt during the Winter of 2016/2017
Source: Viruses. 2019 Oct 27;11(11):990. doi: 10.3390/v11110990 (PMC6893538; doi:10.3390/v11110990)
Supplement: Supplementary file 1 [file viruses-11-00990-s001.pdf]

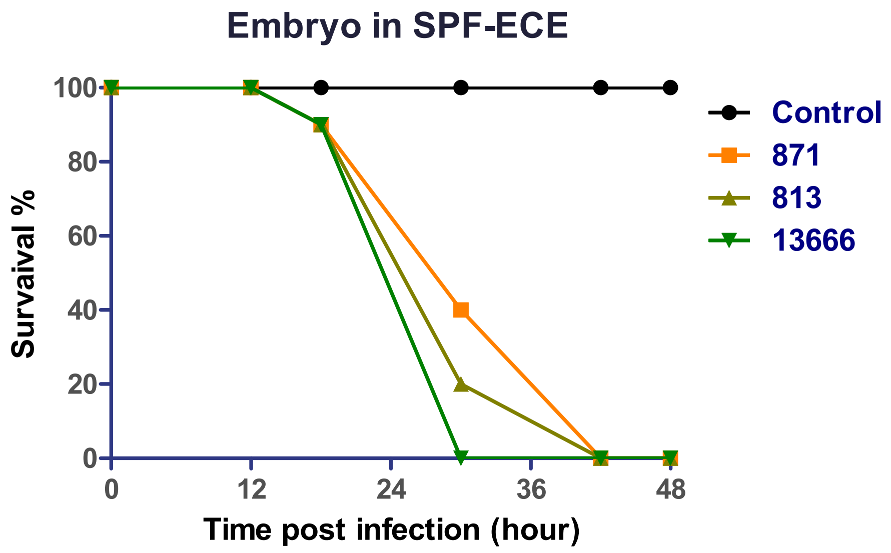

Supplementary Figure 1: Mortality rate of embryos of SPF-ECE infected with different H5N8 viruses

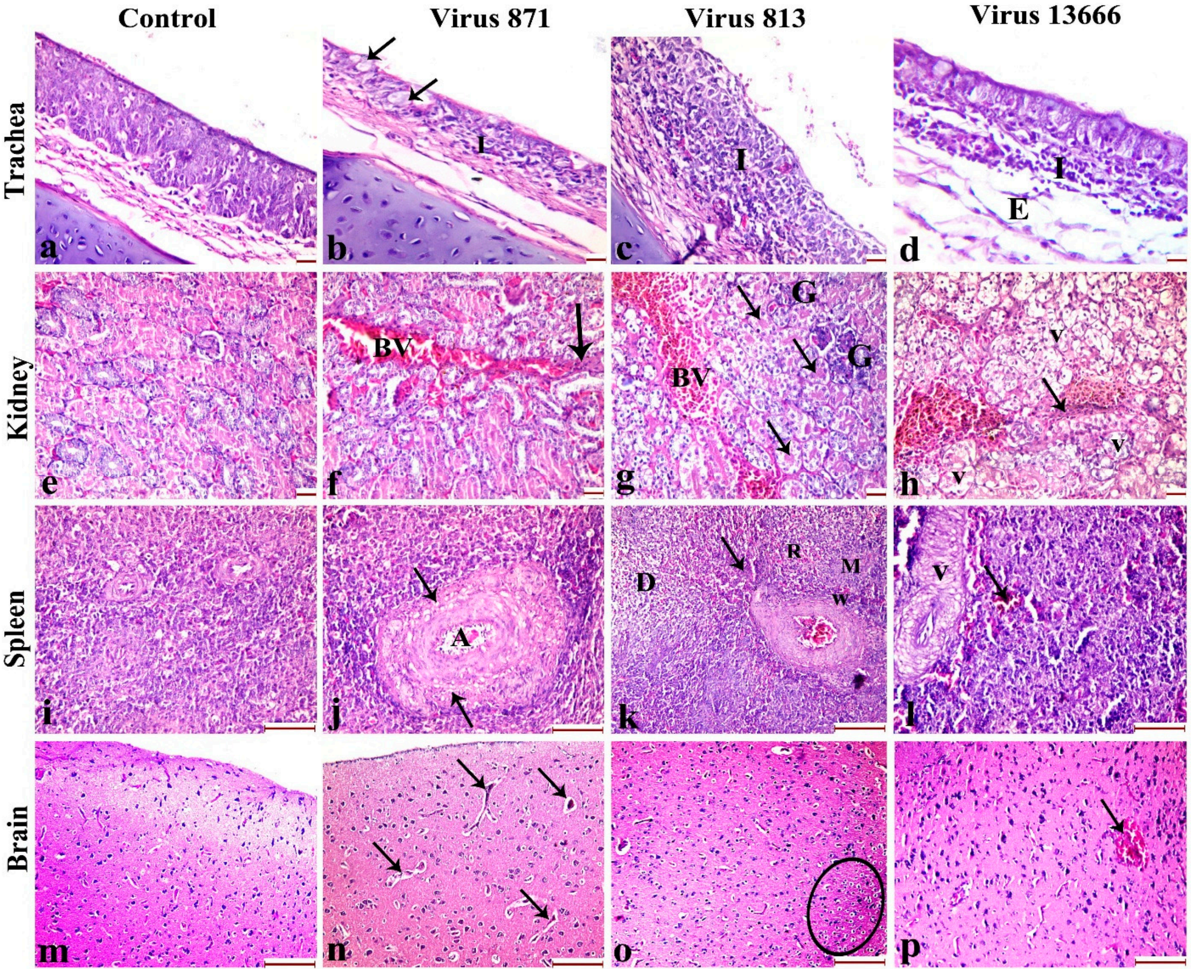

Supplementary Figure S2 : H&E-stained sections of trachea, kidney, spleen, and brain of infected ducks with different H5N8 viruses .H&E-stained sections of (duck trachea a,b,c,d) of (a) control group showing normal histological structure of the trachea with intact pseudostratified columnar ciliated epithelium and lamina propria (b) virus 871 showing activation of mucous secreting glands (arrows) and mild lymphocytic infiltration (I), (b) virus 813 showing extensive lymphocyte reaction where the mucosa and submucosa are completely flooded with dense lymphocytic infiltration (I), (c) virus 13666 showing edema (E) of the lamina propria together with diffuse infiltration (I) of the submucosa with lymphoid cells.

(duck kidney e,f,g,h) (e) control group showing normal glomeruli and tubules, (f) virus 871 showing marked dilatation and congestion of blood vessels (BV). Note: lymphoid inflammatory infiltration (arrow), (g) virus 813 showing distortion of the glomeruli (G) with obliteration of the Bowman's spaces, tubular casts (arrows) and marked vascular dilatation and congestion (BV), (h) virus 13666 showing extensive vacuolation (V) of the tubular epithelial lining with marked vascular congestion, blood extravasation and perivascular cuffing of inflammatory cells (arrow).

(duck spleen i,j,k,l) (i) control group showing normal architecture of the red and white pulps of the spleen, (j) virus 871 showing marked hypertrophy of the central artery (A) with vacuolation and

disintegration of its thickened muscle layer (arrows), (k) virus 813 showing some areas with highly condensed and closely packed lymphocytes (arrow) and other areas with lymphocyte depletion (D). M: marginal zone between the white pulp (W) and the red pulp (R), (k) virus 13666 showing extensive vacuolation (V) involving the entire circumference of the arteriolar wall. Note: dilated sinusoids (arrow) and ill-defined marginal zone between the white pulp and the red pulp.

(duck cerebral cortex m,n,o,p) (m) control group showing normal architecture of the cerebral cortex, (n) virus 871 showing dilatation of cerebral blood vessels (arrows) with cerebral oedema (o) virus 813 showing marked astrogliosis and obvious increase in neuroglial cells (circle) (p) virus 13666 showing marked degeneration of cerebral neurons, neurophagia and dilated congested cerebral blood vessel (arrow). (Scale bar 200μm).

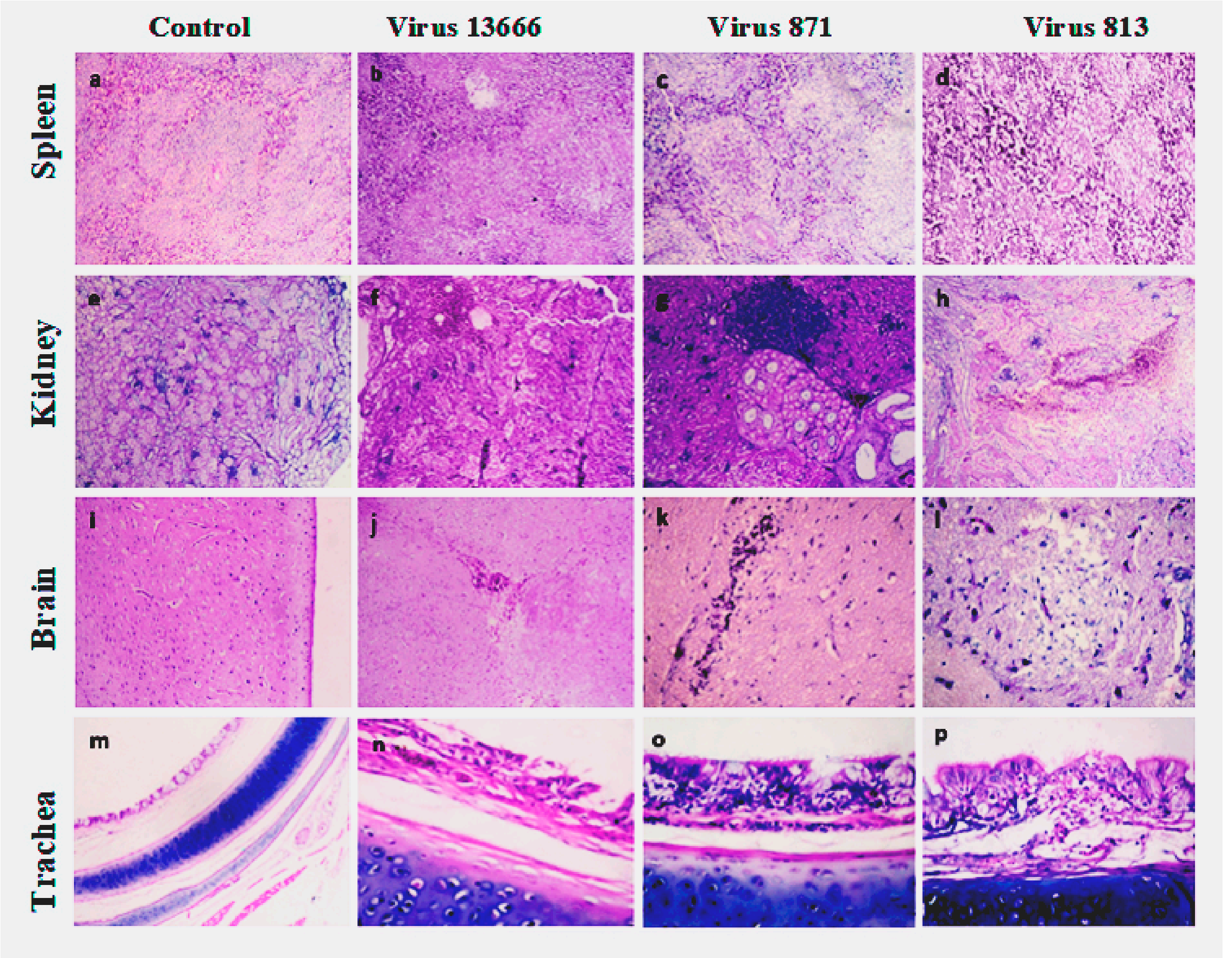

Supplementary Figure S3 : H&E-stained sections of trachea, kidney, spleen, and brain of infected chickens with different H5N8 viruses. H&E-stained sections of (chicken spleen a,b,c,d) X10 (a) Control group showing white and red pulp with normal histological structure., (b) Virus 13666 showing necrosis of the lymphoid elements specially in the white pulp., (c) Virus 871 showing exhaustion of lymphocytes in both white and red pulp., (d) Virus813 showing exhaustion of lymphocytes in both white and red pulp with severe hemorrhage at the red pulp.

(chicken kidney e,f,g,h) X10 (e) Control group showing normal histological structure of the renal cortex and renal medulla. (f) Virus 13666 showing necrosis of the renal tubular epithelium with severe interstitial hemorrhage. (g) Virus 871 showing mild degeneration in the renal tubular epithelium with focal lymphoid cell aggregation in the interstatium (h) Virus813 showing sever necrosis of both renal tubular epithelium and some glomeruli with severe interstitial hemorrhage and lymphoid cell reaction.

(chicken brain i,j,k,l) X10 (i) Control group showing normal histological structure of both white and gray matter in the cerebrum. (j) Virus 13666 showing vascular congestion with perivascular edema and hemorrhage in the cerebral blood vessels. (k) Virus 871 showing small foci of encephalomalacia with glia cell infiltration in the cerebral hemisphere. (l) Virus813 showing large foci of encephalomalacia with glia

cell infiltration in the cerebral hemisphere, most of neurons undergo neuronal degeneration up to necrosis.

(chicken trachea m,n,o,p) X40 (m) Control group showing normal histological structure of tracheal epithelium, lamina propria and cartilage. (n) Virus 13666 showing congestion of the vasculature and degeneration of the epithelial lining the trachea. (o) Virus 871 showing mild tracheitis with degeneration of the epithelial lining, congestion and inflammatory cell infiltration in the lamina propria., (p) Virus813 showing tracheitis with degeneration of the epithelial lining associated with inflammatory edema, congestion and lymphocytic cell infiltration in the lamina propria.

Supplementary Table S1: Nucleotide sequence homology of the 3 forms of Egyptian H5N8 viruses

Segment	871 and 813	871 and 13666	813 and 13666
**PB2**	92.3	97	91.8
**PB1**	99.3	94.5	94.5
**PA**	95.4	95.7	98.6
**HA**	99	98.6	98.5
**NP**	94.9	94.9	97.3
**NA**	99.2	99	99
**M**	98.8	99.1	98.7
**NS**	98.7	99.1	98.7

Supplementary Table S2: Analysis of mammalian virulence determinants in the viral PB2, PB1, PA, NP, M2, NS1, and NS2 proteins

Protein		Virulent	A virulent	871	813	13666	Reference
**PB2**	627	K	E	E	E	E	[1]
	147	L	M	I	I	I	[1]
	250	G	V	V	V	V	[1]
	504	V	I	V	V	V	[2]
	701	N	D	D	D	D	[3]
	591	K	Q	Q	Q	Q	[4]
**PB1**	317	I	M/V	M	M	M	[5, 6]
**PA**	127	V	I	I	V	V	[7]
	672	L	F	L	L	L	[8]
	100	R	V	V	V	V	[9]
	550	L	I	L	L	L	[2]
**NP**	470	R	K	K	K	K	[10]
**M2**	64	S/A/F	P	S	S	S	[7]
	69	P	L	P	P	P	[7]
**NS1**	42	S	A/P	S	S	S	[11]
	92	E	D	D	D	D	[6]
	103	L	F	F	F	F	[12]
	106	I	M	M	M	M	[12]
	189	N	D/G	D	D	D	[13]
**NS2**	31	I	M	M	M	M	[13]
	56	Y	H/L	H	H	H	[13]

Supplementary table S3: Analysis of host range genetic determinants in the PB2, PB1, PA, NP, M1, M2, NS1, and, NS2 proteins in H5N8 viruses.

Viral protein	aa site	Avian preference	Mammalian preference	871	813	13666	Reference
**PB2**	44	A	S	A	A	A	[5, 14]
	64	M	T	M	M	M	[15]
	81	T	M	T	T	T	[14]
	199	A	S	A	A	A	[5, 14]
	591	Q	K	Q	Q	Q	[4]
	627	E	K	E	E	E	[1]
	661	A	T	A	A	A	[16]
	667	V	I	I	V	V	[17, 18]
	701	D	N	D	D	D	[3]
	702	K	R	K	K	K	[16]
**PB1**	13	L	P	P	P	P	[19]
	336	V	I	V	V	V	[5]
	375	N	S	N	N	N	[20]
PB1-F2	73	K	R	K	K	K	[17]
	79	R	Q	R	R	R	[17, 21]
	82	L	S	L	L	L	[17, 21]
**PA**	28	P	L	P	P	P	[22]
	55	D	N	D	D	D	[5, 14]
	57	R	Q	R	Q	R	[5]
	100	V	A	V	V	V	[23]
	133	E	G	E	E	E	[24]
	225	S	C	S	S	S	[25]
	241	C	Y	C	C	C	[26]
	268	L	I	L	L	L	[25]
	356	K	R	K	K	K	[5]
	382	E	D	E	E	E	[14, 27]
	404	A	S	A	A	A	[5]
	409	S	N	S	S	S	[5, 14]
	552	T	S	T	T	T	[25]
	615	K	L	K	K	K	[28]
NP	33	V	I	V	V	V	[5, 14]
	16	G	D	S	G	G	[29]
	61	I	L	I	I	I	[14, 25]
	109	I	V	I	I	I	[5]
	136	L	M	L	L	L	[14]
	214	R	K	R	R	R	[5, 14]
	313	F	Y	F	F	F	[5, 14]
	357	Q	K	Q	Q	Q	[5]
	372	E	D	E	E	E	[5]

	398	K	Q	Q	Q	Q	[5]
	455	D	E	D	D	D	[5]
**M1**	15	V	I	V	V	V	[30]
	115	V	I	V	V	V	[25]
	121	T	A	T	T	T	[25]
	137	T	A	T	T	T	[14, 25]
**M2**	11	T	I	T	T	T	[5]
	16	E	G/D	E	E	E	[14]
	20	S	N	S	S	S	[5, 14]
	28	I	I/V	I	I	I	[14]
	57	Y	H	Y	Y	Y	[5]
	55	L	F	L	L	L	[31]
	86	V	A	V	V	V	[17]
**NS1**	227	E	K/R	G	-	G	[32]
**NEP**	70	S	G	G	G	G	[17, 18]

Supplementary table S4: Amino acid variations among the 3 forms of Egyptian H5N8 Viruses

Viral protein	Site	871	813	13666
**PB2**	187	K	R	R
	255	V	I	V
	288	Q	H	Q
	292	V	I	V
	340	K	R	R
	346	P	T	T
	356	I	V	V
	398	V	I	I
	451	I	V	I
	474	S	S	T
	630	K	R	R
	667	I	V	V
	680	D	D	E
	684	S	A	A
	707	A	A	S
	714	S	S	G
**PB1**	110	A	A	T
	152	S	S	L
	168	R	R	K
	221	A	A	V
	225	N	N	D
	229	K	K	I
	232	E	E	V
	292	N	N	Y
	298	P	L	L
	374	T	T	A
	438	W	W	G
	552	T	I	I
	574	F	F	S
	595	G	G	E
	619	D	D	T
	621	Q	Q	P
	622	G	G	D
	626	N	N	M
	628	L	L	R
	631	F	F	L
	680	R	R	K

	694	G	S	S
**PB1-F2**	42	c	Y	Y
	46	T	M	T
	48	Q	Q	R
	54	L	L	Q
	66	N	N	S
	71	S	Y	S
	74	T	T	I
	81	K	K	R
	85	K	K	R
	length	52	52	90
**PA**	208	A	T	T
	211	M	M	L
	345	I	L	L
	367	K	R	K
**PA-X**	27	D	D	N
	57	R	Q	R
	59	E	G	G
	96	N	H	H
	101	D	E	D
	127	I	V	V
	183	A	A	V
	184	S	N	N
	207	S	L	L
**HA**	15	K	E	K
	191	L	L	M
	284	E	G	E
	503	D	Y	D
	516	E	E	G
	532	P	L	L
**NP**				
	27	A	A	V
	105	M	V	V
	318	P	P	S
	423	TA	AT	AT
	452	R	R	K
**NA**	8	V	A	V
	10	V	I	I
	44	I	I	T
	79	E	E	D
	88	P	P	T

	89	I	I	V
	125	V	V	A
	311	K	R	R
	342	Q	Q	L
	387	R	Q	Q
	390	R	K	R
	462	I	V	I
**M1**	33	A	V	A
	85	N	S	N
	130	I	L	L
	208	Q	K	Q
	248	L	M	L
**M2**	18	N	K	N
	95	E	E	G
	96	L	L	S
**NS1**	82	A	A	D
	165	S	S	F
	205	S	N	S
	214	L	F	L
	215	P	S	P
**NS2**	48	A	T	A
	82	E	E	G

References

1. Wang, J.; Sun, Y.; Xu, Q.; Tan, Y.; Pu, J.; Yang, H.; Brown, E. G.; Liu, J., Mouse-adapted H9N2 influenza A virus PB2 protein M147L and E627K mutations are critical for high virulence. *PLoS One* **2012,** 7, (7), e40752.
2. Rolling, T.; Koerner, I.; Zimmermann, P.; Holz, K.; Haller, O.; Staeheli, P.; Kochs, G., Adaptive mutations resulting in enhanced polymerase activity contribute to high virulence of influenza A virus in mice. *J Virol* **2009,** 83, (13), 6673-80.
3. Teng, Q.; Zhang, X.; Xu, D.; Zhou, J.; Dai, X.; Chen, Z.; Li, Z., Characterization of an H3N2 canine influenza virus isolated from Tibetan mastiffs in China. *Veterinary Microbiology* **2013,** 162, (2), 345-352.
4. Mok, C. K. P.; Lee, H. H. Y.; Lestra, M.; Nicholls, J. M.; Chan, M. C. W.; Sia, S. F.; Zhu, H.; Poon, L. L. M.; Guan, Y.; Peiris, J. S. M., Amino Acid Substitutions in Polymerase Basic Protein 2 Gene Contribute to the Pathogenicity of the Novel A/H7N9 Influenza Virus in Mammalian Hosts. *Journal of Virology* **2014,** 88, (6), 3568-3576.
5. Chen, G. W.; Chang, S. C.; Mok, C. K.; Lo, Y. L.; Kung, Y. N.; Huang, J. H.; Shih, Y. H.; Wang, J. Y.; Chiang, C.; Chen, C. J.; Shih, S. R., Genomic signatures of human versus avian influenza A viruses. *Emerg Infect Dis* **2006,** 12, (9), 1353-60.
6. Lee, M. S.; Deng, M. C.; Lin, Y. J.; Chang, C. Y.; Shieh, H. K.; Shiau, J. Z.; Huang, C. C., Characterization of an H5N1 avian influenza virus from Taiwan. *Vet Microbiol* **2007,** 124, (3-4), 193-201.
7. Lycett, S. J.; Ward, M. J.; Lewis, F. I.; Poon, A. F.; Kosakovsky Pond, S. L.; Brown, A. J., Detection of mammalian virulence determinants in highly pathogenic avian influenza H5N1 viruses: multivariate analysis of published data. *J Virol* **2009,** 83, (19), 9901-10.
8. Li, Z.; Chen, H.; Jiao, P.; Deng, G.; Tian, G.; Li, Y.; Hoffmann, E.; Webster, R. G.; Matsuoka, Y.; Yu, K., Molecular basis of replication of duck H5N1 influenza viruses in a mammalian mouse model. *Journal of virology* **2005,** 79, (18), 12058-64.
9. Otte, A.; Sauter, M.; Daxer, M. A.; McHardy, A. C.; Klingel, K.; Gabriel, G., Adaptive Mutations That Occurred during Circulation in Humans of H1N1 Influenza Virus in the 2009 Pandemic Enhance Virulence in Mice. *Journal of Virology* **2015,** 89, (14), 7329-7337.
10. Chen, L.; Wang, C.; Luo, J.; Li, M.; Liu, H.; Zhao, N.; Huang, J.; Zhu, X.; Ma, G.; Yuan, G.; He, H., Amino Acid Substitution K470R in the Nucleoprotein Increases the Virulence of H5N1 Influenza A Virus in Mammals. *Front Microbiol* **2017,** 8, 1308.
11. Jiao, P.; Tian, G.; Li, Y.; Deng, G.; Jiang, Y.; Liu, C.; Liu, W.; Bu, Z.; Kawaoka, Y.; Chen, H., A single-amino-acid substitution in the NS1 protein changes the pathogenicity of H5N1 avian influenza viruses in mice. *Journal of virology* **2008,** 82, (3), 1146-54.
12. Dankar, S. K.; Wang, S.; Ping, J.; Forbes, N. E.; Keleta, L.; Li, Y.; Brown, E. G., Influenza A virus NS1 gene mutations F103L and M106I increase replication and virulence. *Virology journal* **2011,** 8, (1), 13.
13. Subbarao, K.; Shaw, M. W., Molecular aspects of avian influenza (H5N1) viruses isolated from humans. *Reviews in medical virology* **2000,** 10, (5), 337-48.
14. Shaw, M.; Cooper, L.; Xu, X.; Thompson, W.; Krauss, S.; Guan, Y.; Zhou, N.; Klimov, A.; Cox, N.; Webster, R.; Lim, W.; Shortridge, K.; Subbarao, K., Molecular changes associated with the transmission of avian influenza a H5N1 and H9N2 viruses to humans. *Journal of medical virology* **2002,** 66, (1), 107-14.
15. Guilligay, D.; Tarendeau, F.; Resa-Infante, P.; Coloma, R.; Crepin, T.; Sehr, P.; Lewis, J.; Ruigrok, R. W.; Ortin, J.; Hart, D. J.; Cusack, S., The structural basis for cap binding by influenza virus polymerase subunit PB2. *Nature structural & molecular biology* **2008,** 15, (5), 500-6.
16. Kuzuhara, T.; Kise, D.; Yoshida, H.; Horita, T.; Murazaki, Y.; Nishimura, A.; Echigo, N.; Utsunomiya, H.; Tsuge, H., Structural Basis of the Influenza A Virus RNA Polymerase PB2 RNA-binding Domain Containing the Pathogenicity-determinant Lysine 627 Residue. *Journal of Biological Chemistry* **2009,** 284, (11), 6855-6860.
17. Chen, G.-W.; Chang, S.-C.; Mok, C.-k.; Lo, Y.-L.; Kung, Y.-N.; Huang, J.-H.; Shih, Y.-H.; Wang, J.-Y.; Chiang, C.; Chen, C.-J.; Shih, S.-R., Genomic signatures of human versus avian influenza A viruses. *Emerg Infect Dis* **2006,** 12, (9), 1353-1360.

[illegible] viruses. *Scientific Reports* **2014,** 4, 7455.
20. Jeffery K. Taubenberger, A. H. R., Raina M. Lourens, Ruixue Wang, Guozhong Jin and Thomas G. Fanning, Characterization of the 1918 influenza virus polymerase genes. *nature* **2005,** 437, (6), 889-893.
21. Alymova, I. V.; Green, A. M.; van de Velde, N.; McAuley, J. L.; Boyd, K. L.; Ghoneim, H. E.; McCullers, J. A., Immunopathogenic and antibacterial effects of H3N2 influenza A virus PB1-F2 map to amino acid residues 62, 75, 79, and 82. *J Virol* **2011,** 85, (23), 12324-33.
22. Wanitchang, A.; Jengarn, J.; Jongkaewwattana, A., The N terminus of PA polymerase of swine-origin influenza virus H1N1 determines its compatibility with PB2 and PB1 subunits through a strain-specific amino acid serine 186. *Virus research* **2011,** 155, (1), 325-333.
23. Wang, D.; Tang, G.; Huang, Y.; Yu, C.; Li, S.; Zhuang, L.; Fu, L.; Wang, S.; Li, N.; Li, X.; Yang, L.; Lan, Y.; Bai, T.; Shu, Y., A returning migrant worker with avian influenza A (H7N9) virus infection in Guizhou, China: a case report. *Journal of Medical Case Reports* **2015,** 9, (1), 109.
24. Brown, E. G.; Liu, H.; Kit, L. C.; Baird, S.; Nesrallah, M., Pattern of mutation in the genome of influenza A virus on adaptation to increased virulence in the mouse lung: Identification of functional themes. *Proceedings of the National Academy of Sciences of the United States of America* **2001,** 98, (12), 6883-6888.
25. Finkelstein, D. B.; Mukatira, S.; Mehta, P. K.; Obenauer, J. C.; Su, X.; Webster, R. G.; Naeve, C. W., Persistent Host Markers in Pandemic and H5N1 Influenza Viruses. *Journal of Virology* **2007,** 81, (19), 10292-10299.
26. Yamaji, R.; Yamada, S.; Le, M. Q.; Ito, M.; Sakai-Tagawa, Y.; Kawaoka, Y., Mammalian Adaptive Mutations of the PA Protein of Highly Pathogenic Avian H5N1 Influenza Virus. *Journal of Virology* **2015,** 89, (8), 4117-4125.
27. Taubenberger, J. K.; Reid, A. H.; Lourens, R. M.; Wang, R.; Jin, G.; Fanning, T. G., Characterization of the 1918 influenza virus polymerase genes. *Nature* **2005,** 437, (7060), 889-893.
28. Gabriel, G.; Dauber, B.; Wolff, T.; Planz, O.; Klenk, H.-D.; Stech, J., The viral polymerase mediates adaptation of an avian influenza virus to a mammalian host. *Proceedings of the National Academy of Sciences of the United States of America* **2005,** 102, (51), 18590-18595.
29. Lipatov, A. S.; Yen, H.-L.; Salomon, R.; Ozaki, H.; Hoffmann, E.; Webster, R. G., The role of the N-terminal caspase cleavage site in the nucleoprotein of influenza A virus in vitro and in vivo. *Archives of Virology* **2008,** 153, (3), 427-434.
30. Katz, J. M.; Lu, X.; Tumpey, T. M.; Smith, C. B.; Shaw, M. W.; Subbarao, K., Molecular correlates of influenza A H5N1 virus pathogenesis in mice. *Journal of virology* **2000,** 74, (22), 10807-10.
31. Pan, C.; Jiang, S., E14-F55 combination in M2 protein: a putative molecular determinant responsible for swine-origin influenza A virus transmission in humans. *PLoS currents* **2009,** 1, RRN1044.
32. Soubies, S. M.; Volmer, C.; Croville, G.; Loupias, J.; Peralta, B.; Costes, P.; Lacroux, C.; Guerin, J. L.; Volmer, R., Species-specific contribution of the four C-terminal amino acids of influenza A virus NS1 protein to virulence. *Journal of virology* **2010,** 84, (13), 6733-47.
